# Pressure in the spotlight: effects of monitoring pressure and outcome pressure on time-sharing performance

**DOI:** 10.1186/s41235-025-00697-4

**Published:** 2026-01-15

**Authors:** Niki Pennanen, Lauri Oksama

**Affiliations:** 1 Department of Computer Science, Aalto University, Espoo, Finland; 2 Department of Psychology and Speech-Language Pathology, University of Turku, Turku, Finland; 3 Human Performance Division, Finnish Defence Research Agency, Riihimäki, Finland

**Keywords:** Attentional control, Time-sharing, Multitasking, Eye movements, Performance pressure, Monitoring pressure, Outcome pressure

## Abstract

Performing under pressure, particularly in multitasking environments, is a critical challenge in both everyday life and high-stakes professions. This study investigated the differential effects of monitoring and outcome pressure on time-sharing performance and allocation of visual attention. Using a within-subjects design, 30 participants completed a recently devised time-sharing task requiring prioritization under three different pressure conditions. We hypothesized that in a high-demand time-sharing environment, outcome pressure would impair task performance and visual sampling of subtasks more than monitoring pressure. To investigate, we recorded participants’ task performance metrics and eye movements. However, our confirmatory analyses found no evidence supporting either hypothesis. In contrast, our additional exploratory analyses revealed that monitoring pressure, not outcome pressure, led to a statistically significant performance decrease. Notably, this effect occurred without changes in visual sampling. This unexpected finding reflects the high sensorimotor demands of the task, specifically the need for precise and rapid mouse movements, which may have been disrupted by the participants’ heightened self-consciousness under monitoring pressure. Our findings contribute to the literature on the differential effects of monitoring and outcome pressure, with potential implications for high-stakes domains like military operations. In situations requiring fine motor control—such as piloting aircraft or operating drones—monitoring pressure may disrupt performance, even without altering attentional allocation. Similarly, everyday activities like driving under observation (e.g., driving tests) or performing in front of an audience may be affected. Understanding how pressure disrupts performance in such scenarios can inform training and support strategies to mitigate its impact.

## Significance statement

Performing multiple tasks under pressure is a challenge faced in various high-stakes environments, including military operations, aviation, healthcare, and professional sports, as well as in everyday life. Psychological pressure can undermine performance, but not all types of pressure affect individuals in the same way. This study investigates how monitoring pressure (being observed or evaluated) and outcome pressure (focus on achieving specific goals) differentially influence multitasking performance and attentional control. By combining task performance metrics with eye-tracking data, our research explores the underlying mechanisms of these effects.

Our findings reveal that monitoring pressure, rather than outcome pressure, may impair performance, particularly in tasks requiring high sensorimotor precision. This disruption seems to occur without changes in attentional allocation, suggesting that self-consciousness under observation can interfere with motor execution. This understanding of pressure effects advances theories of cognitive and motor performance under stress and can inform strategies to mitigate performance decrements in real-world applications.

The potential implications are far-reaching. In military contexts, operators of drones, surveillance systems, or critical weapon platforms may experience reduced accuracy under monitoring pressure. In healthcare, surgeons and medical staff working under observation may face similar challenges. Additionally, these insights extend to everyday situations, such as test-taking, driving evaluations, or performing in front of an audience. By identifying how specific types of pressure disrupt performance, this work can contribute to designing training programs, support strategies, and technological aids to enhance resilience and efficiency in critical settings.

## Introduction

In today’s fast-paced and demanding world, the ability to perform multiple tasks simultaneously in rapid and dynamic environments has become increasingly important. Consider the demands faced by surgeons in operating rooms, drivers at busy intersections, or chefs in restaurants who juggle multiple orders while maintaining quality. These challenges are magnified in high-stakes military contexts, where fighter traffic controllers coordinating missions, medics attending to multiple casualties, or other military operators managing complex systems must maintain exceptional prioritization and multitasking capabilities under significant psychological pressure.

Performing under pressure is not just a concern for athletes or other high-stakes professions; it is also an essential part of daily life. Psychological pressure, stemming from time constraints, high expectations, being evaluated, or complex environments, can significantly impact cognitive processes such as attentional control and time-sharing. This pressure can lead to the phenomenon known as “choking”, where an individual performs significantly below their true skill level (Beilock & Gray, 2007). Attentional control, the ability to focus cognitive resources on relevant stimuli while ignoring distractions, is critical for efficient task performance (Eysenck et al., 2007). It is also crucial for time-sharing, the ability to allocate time and attention between concurrent tasks, which is a fundamental component of multitasking (Wickens, 1991). Understanding how psychological pressure influences these cognitive processes is important not only for theoretical insights but also for practical applications aimed at improving performance in both high-pressure and routine settings.

This study investigates the effects of different categories of psychological pressure on time-sharing performance and the allocation of attention. We employ a demanding time-sharing task and expose participants to two types of pressure, monitoring pressure and outcome pressure, separately. Our focus is to determine through task performance and eye movements whether different types of pressure situations produce distinct effects on performance and attentional processes. The findings have potential applications in military settings, where optimizing performance under stress is vital for success, as well as in other fields such as aviation safety and professional sports.

### Attentional control and time-sharing

Attentional control has been referred to by various terms (Burgoyne & Engle, 2020), including executive control (Baddeley, 1996), cognitive control (Botvinick et al., 2001), and executive attention (Engle, 2002). According to Wickens (2021), attentional control can be understood through two main concepts: the filter of selective attention and the allocation of cognitive resources. The filter allows individuals to concentrate on specific tasks by managing the influx of information, while the resource allocation perspective considers how attentional capacity is distributed among concurrent tasks, especially when task demands are high. Regardless of the terminology or exact underlying definition, attentional control is considered crucial for many complex cognitive tasks.

The related concept of time-sharing is defined as “the process of rapidly switching attention from one task to another when two or more tasks are performed concurrently” (*APA Dictionary of Psychology*, n.d.). Time-sharing is often considered a process that facilitates multitasking, and the terms are frequently used interchangeably. When attempting to perform multiple overlapping tasks simultaneously, the primary challenge is resolving resource conflicts and properly prioritizing tasks. The exact mechanisms by which time-sharing processes resolve these resource problems are debated, with two main views presented. One view suggests that a strong executive component of attention directs focus to the most important tasks (e.g., Baddeley, 1996; Meyer & Kieras, 1997; Norman & Shallice, 1986). The other view, labeled “threaded cognition”, hypothesizes that a specialized executive process is not needed and that different tasks freely negotiate and resolve resource conflicts locally (Salvucci & Taatgen, 2008). For a detailed comparison and empirical study of these views, see Kulomäki et al. (2022).

In this study, we use time-sharing as a platform to investigate how different types of psychological pressure affect performance and allocation of attention during multitasking.

### Mechanisms of pressure

Researchers have long acknowledged the associations between attentional processes and anxiety (e.g., Beck & Clark, 1997; Easterbrook, 1959; Eysenck et al., 2007), often triggered by pressure. High-stakes situations are known to cause performance pressure, which sometimes leads to worse-than-expected outcomes. Traditionally, there have been two different schools of thought to explain why pressure sometimes leads to poor performance in both cognitive and motor tasks (DeCaro et al., 2011). Distraction theories suggest that performance deteriorates under pressure due to task-irrelevant thoughts and worries capturing attention away from the task (Beilock & Carr, 2001; Lewis & Linder, 1997; Wine, 1971). In contrast, explicit monitoring theories argue essentially the opposite: pressure shifts too much attention toward the skill processes and procedures, disrupting their execution (Baumeister, 1984; Beilock & Carr, 2001; R. S. W. Masters, 1992). To address how pressure could both divert attention away from and toward the task at hand, DeCaro et al. (2011) focused on the elements of the pressure situations themselves. While many real-world environments may contain mixed pressure elements, they identified two main categories of performance pressure potentially harming performance: monitoring pressure and outcome pressure.

#### Monitoring pressure

Monitoring pressure arises from performing while being observed and potentially evaluated by others, such as a teacher, audience, or video cameras (DeCaro et al., 2011). This feeling of being watched and evaluated can shift the individual’s focus of attention in automatized tasks toward the skill processes and step-to-step procedures being performed. This can then lead to poorer performance when these processes are typically executed almost automatically, outside of awareness.

This route to failure has strong theoretical foundations in explicit monitoring, or self-focus, theories (e.g., Baumeister, 1984; Beilock & Carr, 2001; R. Masters & Maxwell, 2008; R. S. W. Masters, 1992). Most research on this type of skill failure under pressure has been conducted with sensorimotor skills, but support has also been found in cognitive tasks without motor components (DeCaro et al., 2011). A common feature of these tasks is that they involve highly proceduralized processes requiring little to no attentional control, as they are normally automated to a degree (e.g., Beilock & Carr, 2001; Jackson et al., 2006; R. S. W. Masters, 1992). Overall, monitoring pressure seems to cause “paralysis by analysis”, where constant attempts to control skill execution disrupt normally fluent processes.

This view aligns with hierarchical control models of skilled action, where an outer loop can, under some conditions, monitor the inner loop’s output and thereby interfere with execution by adding discrimination or inhibition demands (Logan & Crump, 2009). Crucially, such monitoring need not alter attentional priorities and, especially in well-learned tasks with available compensatory control, may yield little or no net performance cost.

Therefore, we expect that monitoring pressure will not significantly affect either performance or attentional allocation in our experimental task, which is heavily reliant on conscious attentional control instead of proceduralized processes.

#### Outcome pressure

Outcome pressure can be induced when the individual is offered an incentive to achieve a specific outcome (DeCaro et al., 2011). This can shift attention toward worries and consequences of not achieving the incentivized goal. The incentive does not necessarily have to be monetary; any manipulation that heightens the individual’s metacognitive awareness of the performance situation might lead to rumination on potential outcomes, thereby reducing the attentional resources available for the task. This can effectively transform a single-task situation into a dual-task situation (Beilock & Carr, 2001; Beilock & Gray, 2007).

Outcome pressure’s proposed method of skill failure is rooted in distraction theories (Beilock & Gray, 2007; Eysenck et al., 2007; Wine, 1971). According to Eysenck et al. (2007), anxiety increases the influence of the bottom-up stimulus-driven system at the expense of the top-down goal-driven system. This shift can cause several detrimental effects to task performance, especially when time-sharing between multiple tasks is needed. These effects include reduced inhibition of incorrect responses, increased susceptibility to distractions, and impaired task-switching performance. In a dynamic task environment where constant prioritization is needed, outcome pressure might cause attentional shifts more frequently toward less important targets. Therefore, we expect that in our task, performance will be lower and subtask prioritization will become less optimal under outcome pressure.

#### Previous studies comparing pressures

DeCaro et al. (2011) supported their theories of pressure classification by demonstrating in multiple experiments that outcome pressure-induced distractions when tasks relied heavily on attentional control, while monitoring pressure impaired tasks that functioned best without conscious control. Endres et al. (2020) also found in separate experiments that outcome pressure worsened performances in an inhibition task requiring heavy attentional control, but monitoring pressure had no such effect.

In a rare within-subjects study comparing pressure categories by Soleimani Rad et al. (2022), participants experienced separate conditions for both monitoring and outcome pressure. Their task was to hit table tennis serves as accurately as possible while making quick decisions about the desired type of shot for each ball. The researchers found that decision-making accuracy was worse only under outcome pressure, whereas shot performance was worsened only by monitoring pressure. These results support the hypotheses laid out by DeCaro et al. (2011): cognitive decision-making task requiring attentional control was disrupted by outcome pressure, and the more proceduralized motor task of hitting table tennis balls was disrupted by monitoring pressure.

However, there is also contradictory evidence suggesting that monitoring pressure alone can worsen performance in a classic attentional control task (Belletier et al., 2015). This effect might be particularly pronounced in people with higher working memory capacity, possibly because they have the bandwidth to attend simultaneously to both the task and the presence of evaluative others. Similarly, there is evidence that outcome pressure alone can sometimes worsen performance in simple motor tasks (Geukes et al., 2013), which challenges the hypotheses regarding monitoring and outcome pressure. Thus, more research is needed to better differentiate the effects of these two types of pressure. This is an underrepresented area of research in the field of choking under pressure (Soleimani Rad et al., 2022), and empirical within-subject studies comparing both types of pressure in the same task are particularly rare.

#### Psychological pressure in a military context

In a military context, both outcome pressure and monitoring pressure are highly relevant. Outcome pressure arises in real combat situations, where the results of a soldier’s performance carry serious consequences, such as mission success or the safety of themselves and their fellow service members. For example, dismounted soldiers may engage in an exchange of fire with the enemy, where the outcome of their shooting performance is crucial and may determine survival (for shooting performance under pressure, see e.g., Oudejans, 2008). In aerial combat, a fighter pilot may face the intense psychological and physical demands of a close-range dogfight against an enemy aircraft, where split-second decisions and precise perceptual-motor execution can determine survival. Under such conditions, the weight of potential outcomes amplifies outcome pressure.

In contrast, monitoring pressure is most prominent during training and evaluation, when soldiers are aware that instructors, commanders, or peers are closely observing their actions. It can also arise in certain operational contexts, such as drone piloting, particularly when a military team monitors an operator’s performance. Together, these two types of pressure illustrate how both the stakes of performance outcomes and the experience of being observed can shape psychological responses and operational effectiveness in military settings.

### Goals of the study

Our study makes two main contributions. Firstly, we aim to contribute to the growing body of evidence showing that monitoring pressure and outcome pressure are distinct in how they affect performance and attentional processes, with each pressure type influencing individuals in different ways. This distinction is important for understanding the broader impact of pressure on cognitive and motor tasks. Secondly, we extend this pressure classification to a novel and demanding time-sharing environment, where the specific elements of pressure in multitasking contexts have not been thoroughly explored. By focusing on how time-sharing is affected under different types of pressure, our study provides new insights into how attentional resources are allocated across tasks.

Our experimental task contains four subtasks of varying priority, all requiring only the same visual attentional resources (see Sect. "Stimuli" for task description). The subtasks cannot be performed simultaneously; successful performance requires allocating attention and switching between them dynamically. Previous research on this task found that participants adapt to these varying priorities very quickly and successfully interact with the subtasks according to their importance (Kulomäki et al., 2022). This makes the task an excellent platform for examining the effects of pressure on time-sharing and prioritization.

Because decrements in attentional control can be masked at the performance level by compensatory increases in effort (Eysenck et al., 2007), eye movements are often considered a more sensitive measure of attentional control (e.g., Luo et al., 2017; Wood & Wilson, 2010; Wright et al., 2014). In time-pressured visuomotor tasks, covert attention typically shifts to the saccade target just before the eye moves, and fixation behavior reflects the moment-to-moment outcome of priority-based selection (Deubel & Schneider, 1996; Hoffman & Subramaniam, 1995). Accordingly, gaze provides a high-temporal-resolution proxy for where control is allocated. Therefore, in addition to task performance, we recorded eye movements to evaluate attention allocation using two complementary measures: percentage of viewing time per area of interest (allocation/priority) and visual sampling rate (average dwell time per visit; persistence of engagement).

#### Hypotheses

Our experimental task is designed to require heavy attentional control throughout performance, which should make it susceptible to outcome pressure-induced distractions. Given that the task requires conscious control and does not centrally involve proceduralized skills, we do not expect it to be significantly affected by monitoring pressure.

##### H 1:

 Task performance scores will be lower under outcome pressure (compared to the no-pressure condition) and unaffected by monitoring pressure.

##### H 2:

 Subtask prioritization will become less optimal under outcome pressure but will be unaffected by monitoring pressure. This will be evidenced in eye movements by higher event rate (i.e., more important) subtasks having less average time spent looking at them and them having a lower visual sampling rate, compared to the no-pressure condition. And vice versa, lower event rate subtasks will have more time and a higher visual sampling rate under pressure.

## Methods

The study was conducted in accordance with the Declaration of Helsinki, and the participants were free to withdraw from the study at any time without consequences. The study was approved by the University of Turku’s Ethics Committee for Human Sciences. To support open science, the study was preregistered on the Open Science Framework (OSF) before data collection (https://osf.io/vrj7m/), and all the data used for analyses have been uploaded to our OSF Supplementary (https://osf.io/g4bh5/?view_only=6f3c5fbd80354c21997c698021606c4e).

### Participants

The target sample size was determined using an a priori power analysis with G*Power 3.1 (Faul et al., 2009). The results indicated that to achieve 80% power to detect a medium effect size (*f* =.25) with an alpha level of.05, a total of 28 participants would be needed. Due to our counterbalancing strategy, we required the sample size to be a multiple of six. Therefore, we aimed to recruit 30 participants.

The final sample contains 30 students from the University of Turku who volunteered for the experiment conducted between November 2023 and March 2024. The average age of the 30 participants (24 females, 5 males, and 1 other) was 23.7 years, ranging from 20 to 37 years. Most participants reported being right-handed (26 right-handed and 4 left-handed). Participants were asked about their average weekly video gaming habits: 17 reported playing no video games, 6 reported playing 1 to 5 h weekly, 6 reported playing 6 to 10 h weekly, and 1 reported playing over 20 h weekly. Participants could get both course credits and a 10€ gift voucher for participation. Three experiment sessions were invalidated due to software malfunctions during the sessions and were replaced with new participants to maintain the target sample size of 30.

### Stimuli

To evaluate participants’ attentional control and performance under pressure, we used a computerized time-sharing task originally introduced and described in more detail, including a video sample, by Kulomäki et al. (2022). The task was inspired by the framework of instrument flying, where each instrument uniquely supports the task of flying an airplane, and by the high tempo and demanding multitasking environment characteristic of fighter aviation. However, the design was adapted to be generic and not limited to aviation. The subtasks were purposefully made simple to minimize the influence of experience, skill, and specialized knowledge. The task contained four overlapping subtasks of varying importance, requiring participants to dynamically switch between them. Participants were instructed to focus on all of the tasks without any information given about their priorities or possible strategies. This task paradigm was originally developed by Rantanen (Levinthal & Rantanen, 2004; Rantanen & Levinthal, 2005) and it has also been used successfully in experimental research more recently (M. A. Gray et al., 2023; Kulomäki et al., 2022). The computer task was created with E-Prime (Version 3.0).

**Fig. 1:**
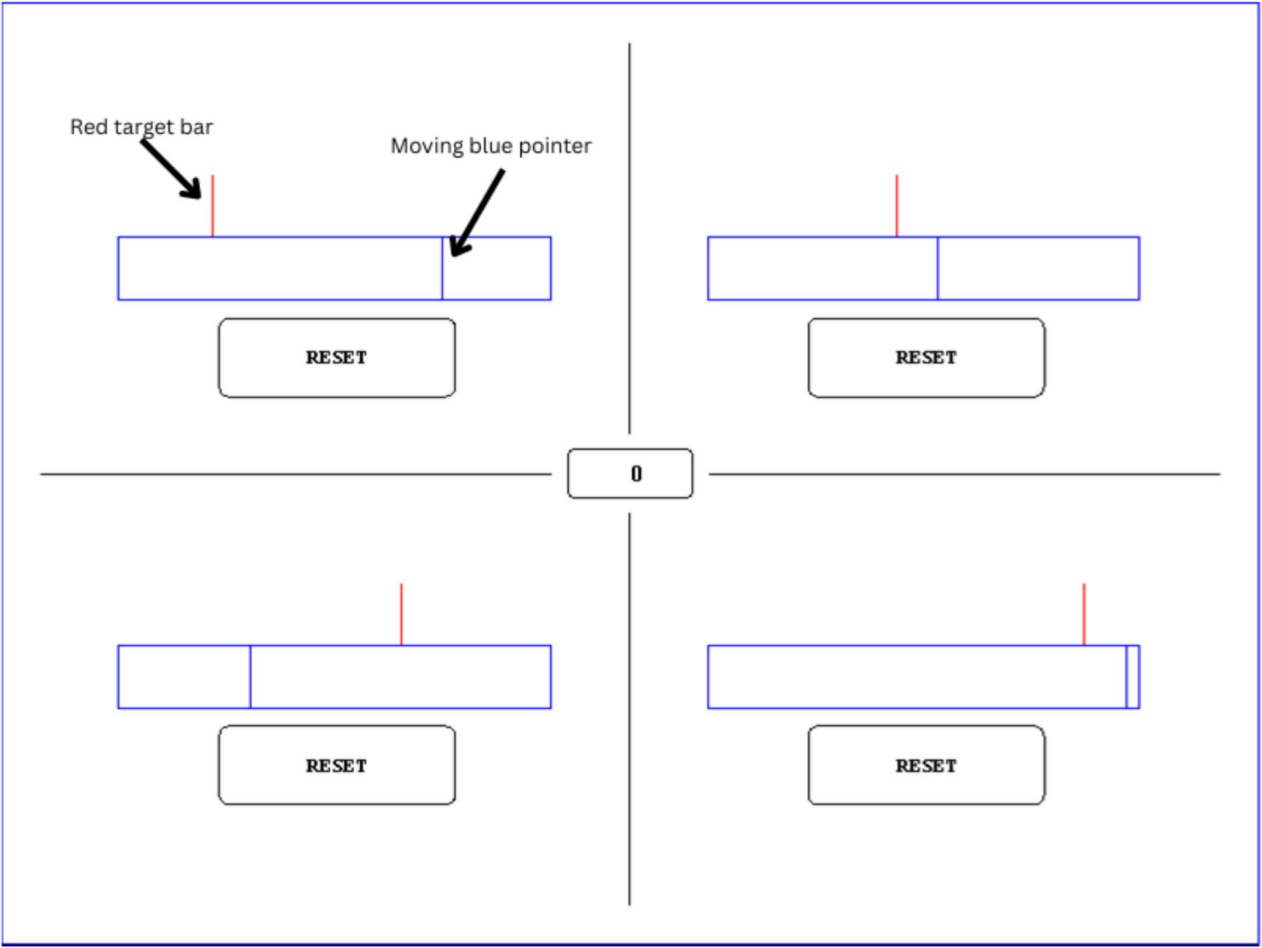
Screenshot from the computer task. The screen is divided into four subtasks of varying importance. The farther to the left the target bar is on the blue frame, the more important the subtask is, as the moving pointer meets with the target bar more frequently. In this figure, the subtask on the top left has the highest importance, and the subtask on the lower right has the lowest importance

As shown in Fig. 1, the screen was split into four quarters, each representing one subtask, with a score counter in the middle. Each subtask contained a blue rectangular frame, with a moving blue pointer within it. Each frame also had a red target bar above it and a reset button below it. During trials, the blue pointer automatically moved at a constant speed from left to right, stopping only at the right edge of the frame. Participants could reset the pointer, returning it to the left edge by pressing the respective reset button with their mouse. The horizontal position of the red target bar (which varied by subtask) determined how often the moving pointer would intersect it, i.e., the subtask’s event rate: bars placed closer to the left edge were encountered more frequently and therefore required more frequent interaction (higher priority). Following the task’s original paradigm (Kulomäki et al., 2022), we set the four predefined event rates at which the pointer meets the target: 0.34, 0.17, 0.11, and 0.08 Hz; subtasks with higher event rates were more important for optimal performance.

During the trials, the participants’ task was to attend to all four subtasks and press the respective reset button as the blue pointer met with the respective red target bar. If the reset button was pressed while the pointer was horizontally within two pixels of the red target bar, the reset was considered successful. A successful reset was signaled by adding 10 points to the score counter in the middle of the screen and by playing a distinct high sound effect. If at the time of the reset the pointer was further than two pixels from the red target, a low sound effect was played, and two points were subtracted from the score counter. Additionally, if a pointer passed the respective target bar without the reset button being pressed, two points were subtracted, and a low sound effect was played once every second until the pointer was reset. The location of the subtasks with different event rates was randomized for each trial.

### Measures

During the computer task, participants’ eye movements were recorded using a desktop-mounted EyeLink 1000 Plus eye tracker. Movements of the right eye were tracked. The eye-movement data was analyzed to identify fixations, which were then assigned to five areas of interest: the four subtasks and the score counter. The main dependent measures were the average percentage of trial time spent looking at different areas of interest, visual sampling rate, and the composite score from the computer task. Visual sampling rate was defined as the total dwell time spent on a subtask divided by the number of gaze visits (enter and leave) to it.

To evaluate the effectiveness of our pressure manipulations, participants self-reported their perceived workload and feelings of anxiety during each condition using the NASA Task Load Index (NASA-TLX; Hart & Staveland, 1988) and the 6-item Spielberger State-Trait Anxiety Inventory (STAI; Marteau & Bekker, 1992; Spielberger, 1970) forms, respectively. Both scales are widely used and validated measures with a long history of usage in experimental research (Hart, 2006; Rossi & Pourtois, 2012). In addition, we measured participants’ pupil size and compared it between conditions. Pupil size has been shown to be an effective objective measurement of mental stress: as stress or anxiety increases, so does the pupil size (e.g., Giannakakis et al., 2022; van der Wel & van Steenbergen, 2018; Yamanaka & Kawakami, 2009).

### Design and procedure

We manipulated one factor: the pressure condition, which had three levels (no-pressure, outcome pressure, and monitoring pressure). In a within-subjects design, each participant experienced all three conditions in a fully counterbalanced order.

First, all participants received verbal and written introductions to the study and provided written informed consent. Crucially, the participants were only informed that the study was about attention and attentional control; they were not aware of the upcoming pressure elements. Before the actual conditions, all participants completed four one-minute practice trials of the computer task to become accustomed to the task. Participants sat 62 cm from the computer screen, and their heads were stabilized using a chinrest. The EyeLink camera was calibrated using a nine-point calibration before each condition, and drift correction was performed before each trial. The calibration error threshold was set at a maximum of 1.0 degrees for a single calibration point and a maximum average of 0.5 degrees for all the points. The lighting in the experiment room was kept constant across all conditions and participants to ensure accurate pupil measurements.

During each condition, after the experimenter gave the possible pressure-inducing instructions, participants filled out a 6-item STAI form (Marteau & Bekker, 1992) before starting the trials, to evaluate their current state anxiety. After completing all trials in a condition, they filled out a NASA-TLX form (Hart & Staveland, 1988), to report their perceived workload during the condition. After all the conditions, the participants were briefly interviewed about their strategies and whether they felt any kind of pressure during the tasks. Finally, everyone was fully debriefed and informed about the deception used during the experiment. The whole session lasted approximately 90 min.

All participants went through the following three conditions in a counterbalanced order.

#### No-pressure condition

The participant completed 10 one-minute trials of the computer task alone in the room. The experimenter left the room and could not see the participant or their computer screen during the trials. There was a 10-s break between trials.

#### Outcome pressure condition

Similarly to the no-pressure condition, the participant completed 10 one-minute trials alone in the room. However, as a deception to induce outcome pressure, the participant was informed before starting the trials that the researchers had predetermined an average score target that all participants should achieve during the experiment. They were told that their current average score in previous trials (practice and possible previous conditions) was lower than the target and thus potentially too low for the researchers to obtain high-quality data from the session.

The participant was encouraged to try improving their performance in the upcoming 10 trials, and that improving their score by 20% compared to their average score so far could earn them a €10 gift voucher for hitting the target. Additionally, the experimenter stated that the participants had been paired beforehand, and both the current participant and their pair would need to achieve the predetermined target for both to earn the gift voucher. The participant was told that their supposed pair had already completed the experiment and achieved the target, leaving it up to the present participant to improve for both individuals to be rewarded.

In reality, there was no pair, and all participants were eligible to receive compensation, regardless of their performance. During the condition, participants were not told their current average score or given a concrete score value to aim for, just that they needed to improve by around 20%. If they asked for the exact target, they were told that it was not revealed yet to encourage them to do their best, not just hit the minimum target. Similar deceptions have been shown to be effective in creating outcome pressure in previous literature (Beilock et al., 2004; DeCaro et al., 2011; Mullen et al., 2016; Smeding et al., 2015; Soleimani Rad et al., 2022).

#### Monitoring pressure condition

As in the other conditions, the participant completed 10 one-minute trials of the computer task. However, this time the experimenter was present in the room, filming the participant and their computer screen with a video camera. The camera and experimenter were positioned behind and to the left of the participant, ensuring that the participant’s computer screen was fully visible to the experimenter. The participant was informed that both they and their screen were being filmed and observed during the task.

The participant was informed that the video might be viewed by other researchers involved in the study to evaluate their performance and that it could additionally be used as presentation material in a future course about psychology lab work. In reality, the camera was only used to induce pressure, and all the video material was immediately deleted without being viewed by anyone. Similar instructions have been shown to be effective at inducing monitoring pressure in previous literature (eg., DeCaro et al., 2011; Endres et al., 2020; Mesagno et al., 2011; Smeding et al., 2015; Soleimani Rad et al., 2022).

## Results

In this section, we first report the subjective and objective measures of the workload and pressure experienced by the participants during the experiment. Next, we present the results of our confirmatory analyses in line with our preregistration. This is followed by additional in-depth exploratory analyses. Assumptions were adequately met for all reported methods. Analyses were conducted with IBM SPSS Statistics (Version 29). All the data used in our analyses is freely available on our online OSF Supplementary.

### Pressure manipulation

To evaluate the effectiveness of our pressure manipulations, we compared the 6-item STAI scores, unweighted NASA-TLX total scores, and pupil size between the no-pressure and pressure conditions. The means and standard deviations are reported in Table 1. In all three instruments, the no-pressure condition had lower scores than both pressure conditions, possibly indicating that participants felt higher pressure and anxiety in the manipulated conditions. However, in paired t-tests, only the differences in pupil size values were statistically significant at the *p* <.05 level (no-pressure vs. monitoring: *t*(29) = − 2.27, *p* =.031, *d* = −0.414; no-pressure vs. outcome: *t*(29) =  − 3.18, *p* =.008, *d* = − 0.58), with a medium effect size. Neither the paired t-tests for STAI (no-pressure vs. monitoring: *t*(29) =  − 0.63, *p* =.534, *d* = − 0.12; no-pressure vs. outcome: *t*(29) =  − 1.95, *p* =.120, *d* = − 0.36) nor NASA-TLX reached statistical significance (no-pressure vs. monitoring: *t*(29) =  − 0.24, *p* =.815, *d* = − 0.43; no-pressure vs. outcome: *t*(29) = − 1.03, *p* =.620, *d* = − 0.19).^1^ Reported *p*-values are adjusted for multiple comparisons using the Holm-Bonferroni method (Holm, 1979).

**Table 1:** Table containing descriptive statistics of measures for evaluating the pressure participants experienced in each experimental condition

Measure	No-pressure		Monitoring pressure		Outcome pressure	
	*M* (*SD*)	95% CI	*M* (*SD*)	95% CI	*M* (*SD*)	95% CI
State anxiety (six-item STAI)	11.50 (2.00)	[10.76, 12.25]	11.73 (2.61)	[10.76, 12.71]	12.40 (3.07)	[11.25, 13.55]
Perceived workload (NASA-TLX)	59.94 (14.50)	[54.53, 65.36]	60.33 (14.94)	[54.75, 65.91]	61.58 (14.73)	[56.08, 67.08]
Pupil size (EyeLink-measured number of pixels)	1016.11 (270.56)	[915.08, 1117.14]	1045.82 (259.81)	[948.80, 1142.83]	1061.15 (279.36)	[956.84, 1165.46]

### Confirmatory analysis

The following confirmatory analyses were conducted as specified in our preregistration. To analyze the effects of pressure on participants’ composite scores in the computer task (Hypothesis 1), we conducted a one-way repeated measures ANOVA with the composite score as the dependent variable and pressure situation as the independent variable with three levels (no-pressure, monitoring pressure, and outcome pressure). To analyze the effects of pressure on participants’ subtask prioritization (Hypothesis 2), we conducted separate repeated measures ANOVAs with the same three pressure levels for two dependent variables: average percentage of trial time spent looking at each subtask and visual sampling rate of each subtask. For both variables, the ANOVAs were repeated for each subtask (0.08 Hz, 0.11 Hz, 0.17 Hz, and 0.34 Hz), resulting in a total of eight separate models.

**Fig. 2:**
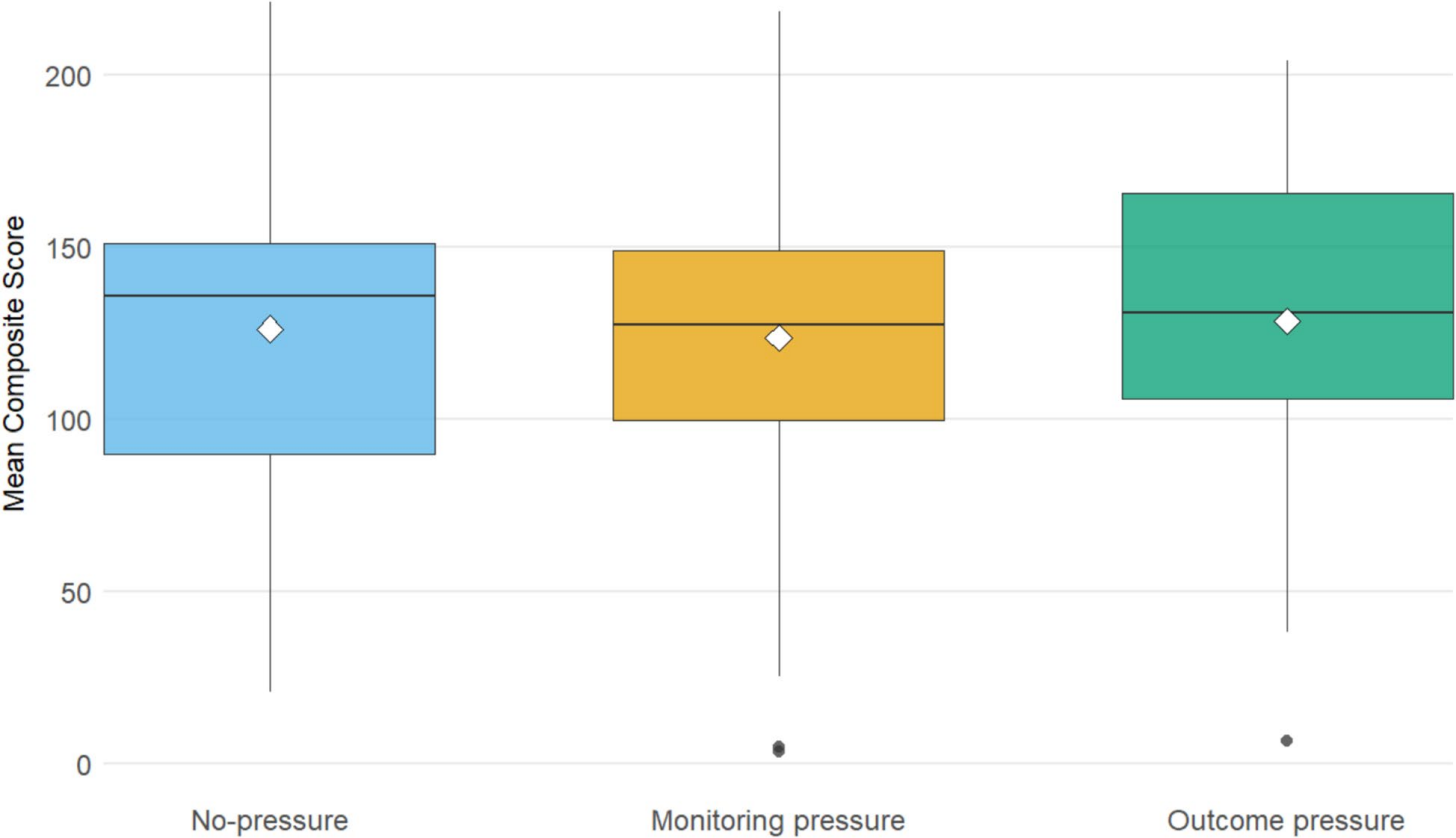
Mean composite scores by condition. White diamonds mark the condition means. For readability, the y-axis is truncated at the 99th percentile, with one outlier beyond the range for Monitoring pressure not shown

The composite score values in different pressure conditions are visualized in Fig. 2. The repeated measures ANOVA revealed no significant differences in score values between pressure conditions (*F*(2, 58) = 0.17, *p* =.845, η_p_^2^ = 0.01). This indicates that being under either kind of pressure did not significantly affect the score values (no-pressure 126.1, monitoring pressure 123.5, and outcome pressure 128.4).

**Fig. 3:**
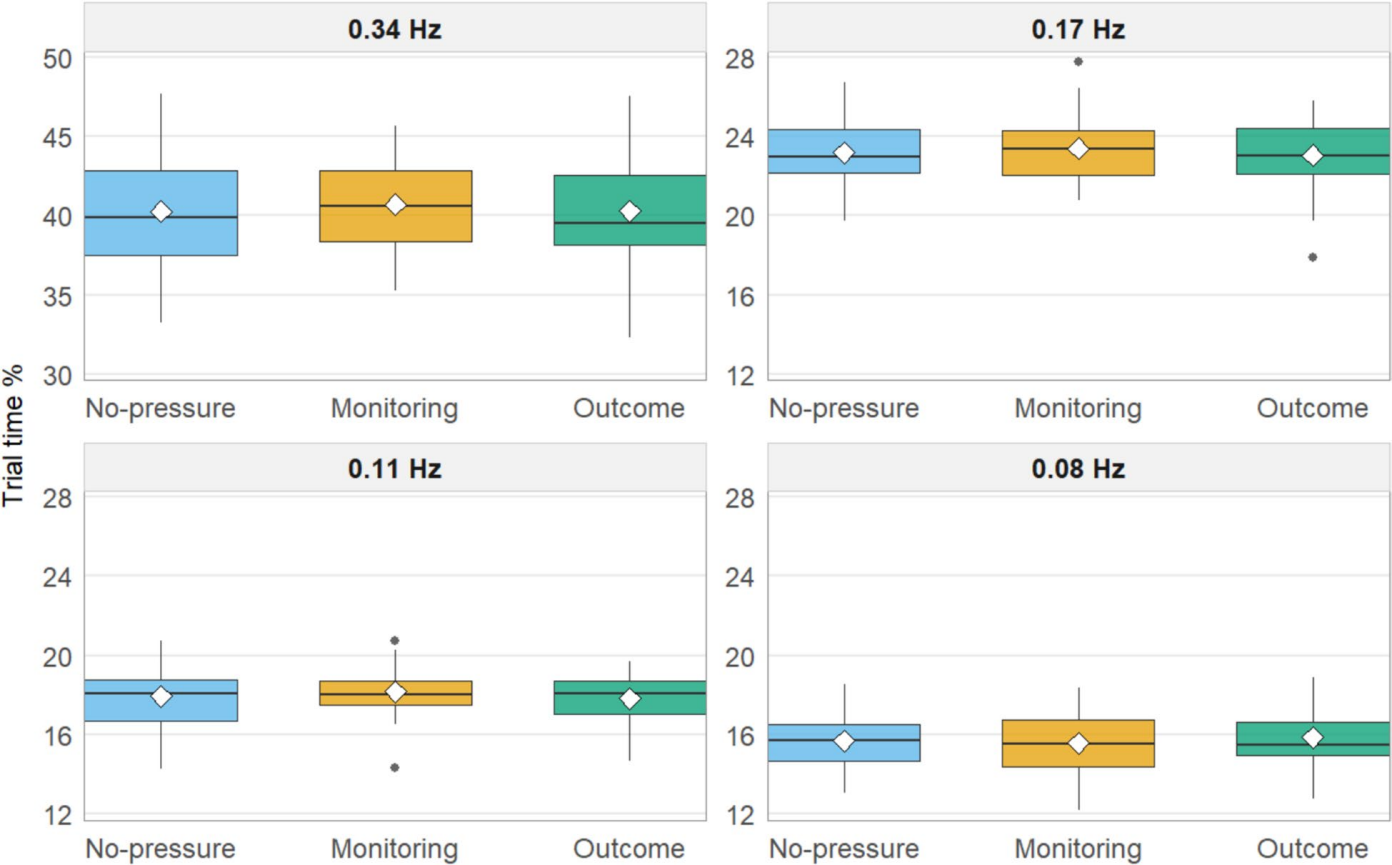
Percentage of trial time spent looking at each subtask, by condition. Panels are ordered by event rate (from highest to lowest). White diamonds mark the condition means. Note: the 0.34 Hz panel uses a different y-axis range for readability

**Fig. 4:**
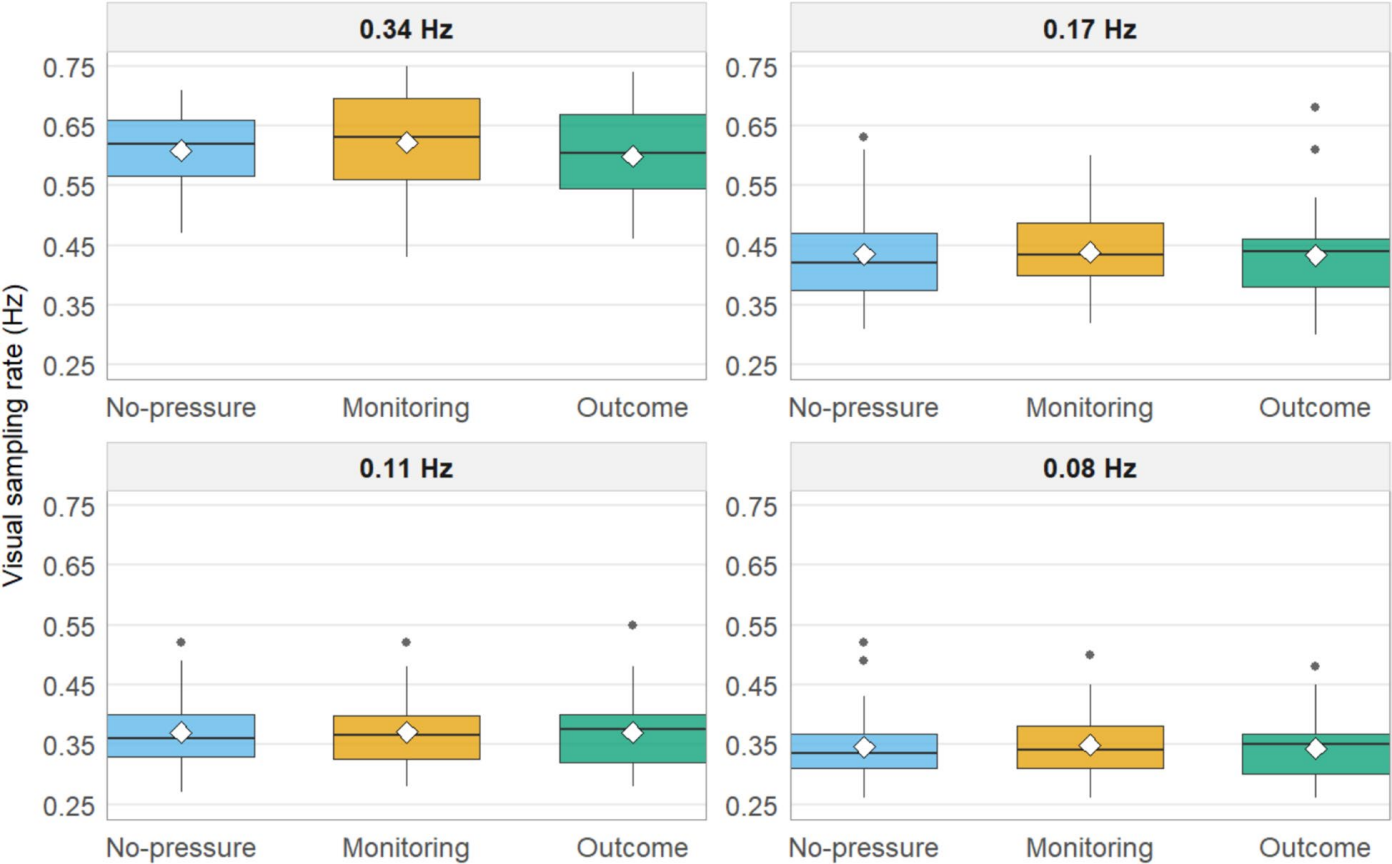
Visual sampling rate (Hz) by condition. Panels are ordered by event rate (from highest to lowest). White diamonds mark the condition means

The percentage of time participants spent looking at each subtask and the visual sampling rates of the subtasks are visualized for each pressure situation in Figs. 3 and 4. The full results of the eight repeated measures ANOVAs are presented in Table 2. None of the models presented statistically significant results. This suggests that participants’ ability to prioritize subtasks seems to have been unaffected by the experienced pressure, and they allocated attention between the subtasks similarly across all conditions.

**Table 2:** Results from the eight repeated measures ANOVAs conducted for the two dependent variables, separately for each subtask, which are identified by their event rates

Dependent variable	Subtask	*F*(2,58)	*p*	η_p_^2^
Percentage trial time spent	0.08 Hz	0.60	.552	0.02
	0.11 Hz	1.04	.359	0.04
	0.17 Hz	0.42	.657	0.01
	0.34 Hz	0.38	.685	0.01
Visual sampling rate	0.08 Hz	0.56	.572	0.02
	0.11 Hz	0.04	.958	0.01
	0.17 Hz	0.21	.812	0.01
	0.34 Hz	0.77	.467	0.03

### Exploratory analysis

In this section, we present our exploratory analyses conducted in addition to the hypothesis testing. The primary goal here is to explore data, uncover patterns, and generate new insights rather than confirm specific hypotheses. Consequently, the *p*-values reported in this section are unadjusted to avoid false negatives, accepting the heightened risk of false positives.

**Fig. 5:**
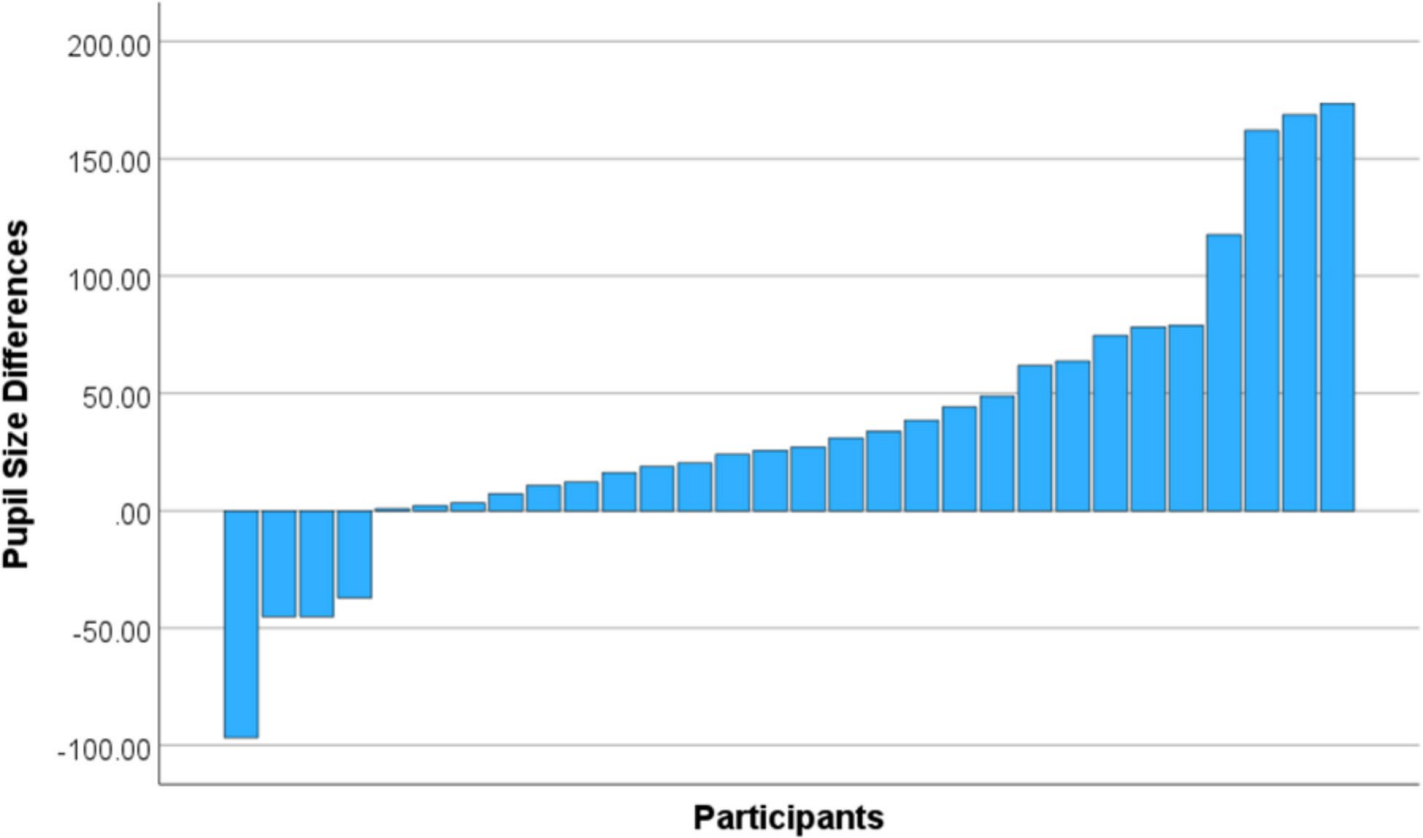
Visualized average changes for each participant’s pupil size while under pressure. Higher positive values indicate that the average pupil size was larger while under pressure

Participants exhibited statistically significant differences in pupil sizes under pressure situations compared to no-pressure conditions. However, in post-experiment interviews, some participants admitted they were somewhat skeptical of the instructions intended to induce pressure. Although none expressed complete disbelief, it is possible that some participants did not feel pressured at all by the manipulations. These individual differences in perceived pressure are also evident in Fig. 5, which shows the average differences in pupil sizes between pressure and no-pressure conditions for each participant. The difference in pupil size for each participant was calculated by taking the average change from the no-pressure condition to the two pressure conditions. We collapsed monitoring and outcome pressures into a single “pressure vs. no-pressure” contrast for the exploratory models to target the common effect of pressure and avoid multiplicity. Specifically, this was computed as the mean of the differences between each pressure condition and the no-pressure condition:

$$Average\, Difference= \frac{\left(Monitoring-No-pressure\right)+(Outcome-No-pressure)}{2}$$

Due to these individual differences, we proceeded to split the dataset in half based on the calculated pupil size differences. We then conducted additional analyses on the half with the highest positive pupil size differences, i.e., the participants for whom the manipulations appeared to be the most effective. Pupil sizes in this high-pressure subset were similar to the complete dataset, with the no-pressure condition having the lowest mean (947.24), monitoring pressure being second highest (1022.43), and finally outcome pressure as the highest (1032.49). Differences in pupil size between no-pressure and both pressure conditions were statistically significant (monitoring: *t*(14) = − 4.24, *p* <.001, *d* = − 1.10; outcome: *t*(14) = − 4.08, *p* <.001, *d* = − 1.05), but the differences between monitoring and outcome pressure were not significant (*t*(14) = − 0.36, *p* =.728, *d* = − 0.09).

To reanalyze the composite score values under pressure, we repeated the previously reported one-way repeated measures ANOVA for the high-pressure subset of the dataset (n = 15). This time, the effect of pressure on task score was statistically significant (*F*(2, 28) = 3.53, *p* =.043, η_p_^2^ = 0.20). Planned contrasts revealed that the average score under monitoring pressure (108.76) was statistically significantly lower than the score under no-pressure (135.78), with a medium effect size (*t*(14) = 2.77, *p* =.015, *d* = 0.72). However, the difference in scores between outcome pressure (127.48) and no-pressure (135.78) was not statistically significant (*t*(14) = 0.80, *p* =.438, *d* = 0.21), and neither were the differences between the two pressure conditions (*t*(14) = 1.69, *p* =.114, *d* = 0.44). Analyses for the average trial time spent looking at different subtasks and visual sampling rates were also repeated on this high-pressure subset of the data, but the results (reported in Table 3) did not differ from those previously reported for the complete dataset.

**Table 3:** Additional analyses conducted for the high-pressure half of the data. Results from the eight repeated measures ANOVAs conducted for time spent looking at each subtask and visual sampling rate, reported separately for each subtask

Dependent variable	Subtask	*F*(2,28)	*p*	η_p_^2^
Percentage trial time spent	0.08 Hz	0.36	.700	0.03
	0.11 Hz	3.14	.059	0.18
	0.17 Hz	1.96	.160	0.12
	0.34 Hz	0.15	.863	0.01
Visual sampling rate	0.08 Hz	0.83	.447	0.06
	0.11 Hz	1.00	.381	0.07
	0.17 Hz	0.01	.995	0.01
	0.34 Hz	0.05	.954	0.01

To investigate what might have caused the lower scores while under monitoring pressure, we then explored the reset errors made by participants, i.e., the instances when they pressed the reset button while the corresponding pointer was over two pixels away from the corresponding target bar. All average reset errors for each experimental condition are presented in Table 4, separately for the whole data and for the high-pressure subset. Repeated measures ANOVA conducted on the complete dataset revealed no significant effect of pressure for the reset error (*F*(2, 58) = 0.17, *p* =.841, η_p_^2^ = 0.01). For the high-pressure subset, the reset errors were on average over 1 pixel higher for both pressure conditions, when compared to the no-pressure condition. Meaning, participants made slightly larger errors while under both pressures. However, the effect of pressure in repeated measures ANOVA for the high-pressure subset was not statistically significant (*F*(2, 28) = 1.95, *p* =.161, η_p_^2^ = 0.12).

**Table 4:** Mean reset errors in different conditions, separately for the whole data and high-pressure half of the data

Condition	Mean reset error in pixels (SD)	
	All data	High-pressure half
No-pressure	8.21 (3.07)	7.47 (2.04)
Monitoring pressure	7.95 (3.22)	8.58 (2.90)
Outcome pressure	8.21 (3.02)	8.49 (2.94)

Next, we classified the reset errors further as early errors and late errors. Early errors were defined as reset button presses happening while the moving pointer was more than two pixels to the left of the target bar. Late errors happened when the moving pointer was more than two pixels to the right of the target bar. The numbers of early and late errors in different conditions are reported in Table 5. Across all conditions and in both the complete data and the high-pressure subset of the data, participants made around 4 to 6 times more late errors than early errors. For the complete data, values for both early and late errors are similar across all conditions. For the high-pressure subset, late errors are similar in all conditions. However, the early errors during monitoring pressure stand out as higher than in other conditions in the high-pressure subset. Repeated measures ANOVA indicates that for the high-pressure subset, the effect of pressure on the number of early errors is not statistically significant, but very close to it (*F*(2, 28) = 3.25, *p* =.054, η_p_^2^ = 0.19). A post hoc paired t-test also indicates that the number of early errors in monitoring pressure is significantly higher compared to no-pressure *t*(14) = 2.41, *p* =.030, *d* = 0.62.

**Table 5:** The number of early and late reset errors in different pressure conditions, separately for the whole data and the high-pressure half of the data

Condition	All data		High-pressure half	
	Early errors	Late errors	Early errors	Late errors
No-pressure	35.33 (27.53)	155.63 (39.18)	27.67 (16.58)	156.47 (40.38)
Monitoring pressure	35.83 (25.92)	153.40 (35.02)	40.33 (24.67)	160.13 (23.61)
Outcome pressure	37.50 (31.71)	153.10 (32.26)	29.93 (16.94)	159.40 (36.08)

## Discussion

The aim of our study was to investigate whether monitoring pressure and outcome pressure exert differing effects on time-sharing performance and allocation of attention. Our first hypothesis predicted that, when compared to the no-pressure condition, outcome pressure would lower task performance scores, while monitoring pressure would have no significant effect. Similarly, our second hypothesis stated that subtask prioritization would deteriorate under outcome pressure but remain unaffected by monitoring pressure. However, our preregistered confirmatory analyses provided no evidence to support either of these hypotheses.

The mean task scores were consistent across all three conditions, with only minor and not statistically significant variations that likely resulted from random chance. A similar pattern emerged for subtask prioritization, as measured by eye movements. Participants allocated their attention and time similarly among the different subtasks across conditions. Consistent with previous research using the same task (Kulomäki et al., 2022), participants appropriately allocated the majority of their time (approximately 40%) to the most important subtask, while dedicating considerably less time (15–23%) to each of the other three subtasks. The visual sampling rate further supports these findings, showing no significant differences in how participants sampled the subtasks, regardless of the experimental condition. Notably, there was no evidence of excessive attentional tunneling toward the most important subtask(s) under pressure. One possible explanation for the absence of focused attention on select subtask(s) under pressure could be the sound effects used in our study. These sound cues alerted participants every second they failed to interact with a subtask, potentially preventing the development of attentional tunneling. It is documented that salient alarms for neglected tasks can mitigate attentional tunneling (Wickens, 2021).

To evaluate the effectiveness of our pressure manipulations, we used both subjective self-report measures and an objective physiological measure: pupil size. While the mean scores for self-reported workload (NASA-TLX) and state anxiety (STAI) were higher in the pressure conditions compared to the no-pressure condition, these differences were not statistically significant. Notably, even in the no-pressure condition, NASA-TLX scores were fairly high, averaging 60 points. Previous meta-analyses have found the mean recorded NASA-TLX scores across hundreds of studies to range between 42 and 49 (Grier, 2015; Hertzum, 2021). For instance, Grier (2015) reported a median score of 46.00 across 31 cognitive task studies, 52.24 across 174 monitoring tasks in different studies, and 52.44 in 24 tasks involving air traffic control. Our participants consistently reported higher values, even in the no-pressure condition. This suggests that our task may be inherently very demanding, potentially limiting the ability of subjective measures like NASA-TLX and STAI to effectively distinguish between our pressure and no-pressure conditions. As the workload and experienced pressure increase toward the extreme ranges, it may become more challenging for participants to subjectively differentiate between the already elevated values.

In contrast to the subjective measures, pupil size was significantly larger in both pressure conditions than in the no-pressure condition, indicating that the manipulations were effective at the group level. As pupil size is very sensitive to lighting changes in the environment, ambient lighting was kept constant across conditions; the only contextual changes were the experimenter’s presence and the camera during monitoring pressure, which did not alter room illumination. The experimental task itself was identical across conditions.

Given heterogeneity in physiological responsiveness, we conducted preregistered analyses on the full sample and then an exploratory follow-up focusing on participants with the largest pupil increases under pressure (median split; “high-pressure” subset). In this subset, task scores were significantly lower under monitoring than no-pressure, whereas the outcome–no-pressure contrast was not significant. This pattern departs from the common expectation that outcome pressure is more disruptive in attention-demanding tasks, though monitoring-related costs have been reported in some contexts (e.g., Belletier et al., 2015). Notably, this difference cannot be explained by greater physiological pressure in monitoring pressure: within the high-pressure subset, outcome pressure elicited an equal or larger pupil response than monitoring (ns).

To explore the reason behind the lower scores under monitoring pressure, we analyzed the specific errors participants made during the task. In our task, each subtask involved a pointer that moved slowly from left to right. Participants had to press a reset button to return the pointer to the left edge of the subtask when the pointer was aligned with a corresponding target bar. They were allowed a leeway of two pixels on either side of the target bar. If participants pressed the reset button when the pointer was more than two pixels to the left of the target bar, they made an early error and lost points. Conversely, if they pressed the reset button when the pointer was more than two pixels to the right of the target bar, they made a late error and lost points.

We first examined the overall magnitudes of reset errors across each condition. In the complete dataset, as expected, the errors were similar across conditions, with only minor random variations. In the high-pressure subset, errors were consistently higher by approximately one pixel in both pressure conditions compared to the no-pressure condition. However, these differences were not statistically significant, and since the error magnitudes were similar under both outcome and monitoring pressure, they could not account for the previously observed differences in task scores between pressure conditions.

Next, we analyzed the frequency of early and late reset errors. In the complete dataset, no significant differences were observed. However, for the high-pressure subset, we found a statistically significant increase in the number of early errors under monitoring pressure compared to the no-pressure condition. No significant differences were found for outcome pressure or late errors. This increase in early errors appears to be the key factor explaining the lower task scores under monitoring pressure. Since there were no differences in the magnitude of errors between pressure conditions, this suggests that participants made slightly smaller errors but did so more frequently under monitoring pressure, leading to a lower overall task score.

A significant portion of explicit monitoring research supporting the theory of monitoring pressure focuses on proceduralized sensorimotor skills, such as golf putting (Beilock & Carr, 2001), baseball batting (R. Gray, 2004), and table tennis (Soleimani Rad et al., 2022). We did not anticipate that monitoring pressure would impair performance in our task, which relies heavily on attentional control rather than proceduralized skills. However, it can be argued that our task does, in fact, involve a significant sensorimotor component: the precise and rapid use of the mouse. The task is highly time-sensitive, requiring quick and accurate mouse usage. Being monitored by the experimenter and recorded by the video camera may have made participants feel that their performance was being closely scrutinized, leading them to become more self-conscious about their mouse movements and clicks. This heightened self-awareness might have caused them to rush their button presses, resulting in early errors and decreased accuracy.

Therefore, we suggest that monitoring pressure impaired task performance not by distracting participants from the task itself but by disrupting the fine motor control task of accurately using the mouse. This aligns with the expected effects of monitoring pressure on sensorimotor tasks and is consistent with hierarchical control accounts: an outer monitoring loop can, under some conditions, introduce additional discrimination and/or inhibition demands on an inner execution loop, interfering with fluency without requiring a change in attentional priorities (Logan & Crump, 2009). The absence of significant changes in gaze-based indices of attentional allocation, together with the higher frequency of early errors, fits this mechanism.

Additionally, it underscores the importance of carefully planning experimental tasks in future research. If monitoring pressure can interfere with the precise use of instruments like a mouse commonly used in computer-based tasks, laboratory experiments investigating psychological pressure should account for this potential execution-level vulnerability in their task design and measurement choices.

However, alternative explanations are also worth considering, since we did not directly measure sensorimotor disruption, and our explanation relies on inferential reasoning. The increase in early errors under monitoring pressure, alongside unchanged gaze allocation, is compatible with a lowered response threshold or reduced inhibitory control account. Attentional control theory (ACT) holds that anxiety impairs goal-directed control by weakening inhibition and shifting resources toward stimulus-driven processing, which can yield premature or imprecise actions (Coombes et al., 2009; Eysenck et al., 2007). Although ACT-type deficits are most often discussed in the context of distraction/anxiety manipulations such as outcome pressure, evaluative monitoring could also elicit anxiety and may tax inhibitory processes; thus, an inhibition-based route remains plausible here. This is also not mutually exclusive with our hierarchical-control framing of monitoring pressure: evaluative self-focus may insert additional discrimination and/or inhibition demands into execution, effectively lowering response thresholds without altering attentional priorities (cf. Logan & Crump, 2009). A follow-up study could directly test this account by including an inhibition assay (e.g., stop-signal or antisaccade) and/or formal response-threshold modeling of click timing alongside the current measures.

Outcome pressure, while not yielding significant results in this study, still probably plays a role in performance failure under different cognitive demands or even with time-sharing performance. Future research should focus on directly measuring the cognitive and motor mechanisms behind performance under both types of pressure. This could include real-time assessments of, e.g., fine motor control, potential rumination behind the distraction while under outcome pressure, cognitive load, and physiological markers of stress (e.g., heart rate variability, electrodermal activity, pupil dilation) to better understand the distinct and potentially interactive effects of monitoring and outcome pressures on task performance.

### Study limitations

Our study has certain limitations that should be acknowledged for consideration in future research. Firstly, while we argue that our pressure manipulations were effective, as evidenced by changes in pupil size, there might be room for further refinement. For outcome pressure, instead of a general request to improve performance, such as the 20% improvement we employed without providing the actual target score, some studies have used specific score targets that participants must achieve (e.g., Mullen et al., 2016). It is possible that a concrete target could increase motivation and introduce more distractions due to the heightened pressure. However, the approach carries potential risks: if the target is set too high, some participants might become discouraged and give up, whereas those who reach the target early might relax for the remainder of the session. Additionally, offering more valuable rewards or enhancing the social pressure aspect, such as by having participants actually see someone they believe is their partner, could potentially increase the effectiveness of the pressure manipulation.

For monitoring pressure, some studies have introduced an additional person into the room during the pressure condition, whose sole purpose is to evaluate the participant's performance (Soleimani Rad et al., 2022). This may exert more pressure on the participant compared to just the presence of the main experimenter and cameras. It should be noted, however, that most other recent pressure studies have used manipulations identical or very similar to ours, with successful results (e.g., DeCaro et al., 2011; Endres et al., 2020; Smeding et al., 2015).

Additionally, the pressure experienced by participants could be measured even more precisely by incorporating further physiological measures known for their accuracy in this domain, such as heart rate variability (Kim et al., 2018) or electrodermal activity (Giannakakis et al., 2022). However, using more physiological sensors comes with the tradeoff of increased invasiveness, which could introduce additional stress and anxiety simply due to the presence of the measuring equipment.

Individual differences among participants could be more thoroughly accounted for in future research. There exists some evidence suggesting that factors such as working memory capacity might influence who is more likely to be distracted under pressure (Beilock & Carr, 2005; Belletier et al., 2015). Additionally, individuals vary in their predisposition to stress, particularly in terms of trait anxiety levels (Eysenck, 1992; Eysenck et al., 2007). Even if these individual differences are not the primary focus of a study, not controlling for them could impact the results. Our sample was also predominantly female, which may limit generalizability, particularly with regard to military samples, which are predominantly male (NATO, 2020).

Some previous research has attempted to distinguish time pressure from other types of pressure (Endres et al., 2020). Our task inherently includes tight time constraints, as is typical in time-sharing environments. If time pressure has distinct effects or significantly interacts with other forms of pressure, its influence might be obscured when combined with other pressure types. Future research should consider this and potentially investigate time pressure as a separate, third type of pressure.

Across measures, effects were heterogeneous: for example, in the physiologically responsive subgroup, monitoring pressure reduced task score without accompanying changes in gaze, whereas outcome pressure elicited a comparable or larger pupil response but no score decrement. This lack of alignment across dependent variables cautions against inferring a unitary “pressure” effect from the present dataset. Therefore, the monitoring-specific decrement should be treated as a tentative, mechanism-level finding (execution interference rather than attentional reallocation) pending replication.

Several of our findings come from exploratory analyses in a modest sample, and as such, should be taken as hypothesis-generating, rather than confirmatory. We stratified participants by pupil-size change (median split) to focus interpretation on those showing a physiological response; this aids interpretability but is only one reasonable approach. A prudent next step could be a preregistered replication with (i) stronger, calibrated pressure manipulations; (ii) systematic measurement and pre-specified mixed-effects modeling of individual-difference moderators (e.g., trait anxiety, stress reactivity, working-memory capacity); and (iii) direct motor-execution measures (e.g., click-timing variability, endpoint accuracy, cursor-trajectory jitter) to test the proposed execution mechanism alongside the existing gaze and score outcomes.

### Conclusions

This study examined the differential effects of monitoring and outcome pressure on time-sharing performance and attentional allocation. Confirmatory analyses did not support the preregistered hypotheses; exploratory analyses suggested a monitoring-specific decrement in task score that is plausibly attributable to execution-level demands (precise mouse use) rather than a reallocation of attention. Effects were heterogeneous across measures (e.g., no gaze changes), so this account is hypothesis-generating and requires replication.

The observed results highlight the complexity of psychological pressure and underscore the importance of considering task-specific factors when designing experiments and interpreting results. Performance pressure, such as mere experimenter presence, could also unintentionally influence, e.g., computer-based tasks in experiments not specifically designed to study or account for pressure. Therefore, researchers should be mindful of the potential for such unintended influences when setting up experimental environments, particularly in studies where precise motor control or attentional focus is critical. The time-sharing and attentional control mechanisms examined in this study also parallel the demands faced by military personnel operating in dynamic and high-pressure environments, where the ability to allocate attention effectively and avoid performance decrements under stress is critical for mission success. In situations requiring fine motor control—such as piloting aircraft or operating drones—monitoring pressure may disrupt performance, even without altering attentional allocation. For example, assembling a large group to observe an operator’s fine motor performance during drone piloting can increase monitoring pressure and impair task execution. Overall, our findings contribute to our understanding of psychological pressure’s effects on performance, with potential implications for enhancing operational effectiveness in military, sports, and other high-stakes domains.

## Data Availability

The datasets generated and analyzed during the current study are available in the OSF repository, https://osf.io/g4bh5/?view_only=6f3c5fbd80354c21997c698021606c4e.

## Footnotes

1 Similarly, exploratory analyses of individual unweighted NASA-TLX dimensions found no significant pairwise differences between any conditions.
